# Advance care directive prevalence among older Australians and associations with person‐level predictors and quality indicators

**DOI:** 10.1111/hex.13264

**Published:** 2021-05-01

**Authors:** Kimberly Buck, Linda Nolte, Marcus Sellars, Craig Sinclair, Ben P. White, Helana Kelly, Ashley Macleod, Karen M. Detering

**Affiliations:** ^1^ Advance Care Planning Australia Austin Health Heidelberg Vic Australia; ^2^ Department of Health Services Research & Policy Research School of Population Health Australian National University ACT Australia; ^3^ Australian Research Council Centre of Excellence in Population Ageing Research University of New South Wales Sydney NSW Australia; ^4^ Neuroscience Research Australia (NeuRA) Sydney Australia; ^5^ School of Psychology University of New South Wales Sydney NSW Australia; ^6^ Australian Centre for Health Research Law Faculty of Law Queensland University of Technology Brisbane Qld Australia; ^7^ Faculty of Health, Arts and Innovation Swinburne University Hawthorn Vic. Australia

**Keywords:** advance care directive, advance care planning, aged care service, general practice, health service, prevalence

## Abstract

**Background:**

Advance care planning (ACP) conversations may result in preferences for medical care being documented.

**Objective:**

To explore the uptake and quality of advance care directives (ACDs) among older Australians accessing health and aged care services, by overall ACP documentation prevalence, person‐level predictors and ACD quality indicators.

**Design and Setting:**

National multi‐centre health record audit in general practices (GP), hospitals and residential aged care facilities (RACF).

**Participants:**

A total of 4187 people aged ≥65 years attending their GP (n = 676), admitted to hospital (n = 1122) or residing in a RACF (n = 2389).

**Main Outcome Measures:**

ACP documentation prevalence by setting and type including person‐completed ACDs and non‐ACD documents (completed by a health professional or someone else); person‐level predictors and quality indicators of ACDs.

**Results:**

Overall ACP documentation prevalence was 46.5% (29.2% weighted). ACD prevalence was 25.3% (14.2% weighted). Unweighted ACD prevalence was higher in RACFs (37.7%) than in hospitals (11.1%) and GPs (5.5%). 35.8% of ACP documentation was completed by a health professional (9.7% weighted), and 18.1% was completed by someone else (10.6% weighted). Having an ACD was positively associated with being female, older, having two or more medical conditions, receiving palliative care, being divorced/separated and being in a RACF. Only 73% of ACDs included full name, signature, document date and witnessing.

**Conclusions and Contribution:**

Low ACP documentation prevalence and a lack of accessible, person‐completed and quality ACDs represent an important ACP implementation issue. Low prevalence is complicated by poor document quality and a higher prevalence of documents being completed by someone other than the person.

## 1 INTRODUCTION

In 2017, there were 56 million deaths globally, with 58% of these deaths recorded for people aged 65 years or older.^1^ While some individuals will die suddenly and unexpectedly, many will experience a prolonged period of deteriorating health, chronic illness, comorbidity and diminishing decision‐making capacity prior to death.^2^ This period is associated with adverse health outcomes, including poorer quality of life,^3^ increased hospitalizations,^4^ unwanted and intensive medical treatments and greater health‐care expenditure.^5^ While almost half of those nearing the end of life will require treatment decisions to be made, the majority will lack the capacity to make their own decisions.^6^, ^7^

Advance care planning (ACP) and related medical treatment legislation enable individuals to maintain choice and control over their health‐care decisions in the event they lose decision‐making capacity in the future. ACP is an on‐going process of reflection and discussion that supports a person to identify and discuss their goals, values and preferences for future care with health‐care providers and loved ones.^8^ The ultimate goal of ACP is that people receive medical care that is consistent with their preferences.^9^

ACP is associated with a range of beneficial outcomes for individuals, their families, health professionals and the health system.^6^, ^10^, ^11^, ^12^, ^13^, ^14^ However, a recent Australian study of people with cancer and support people found that ACP and assistance with the completion of ACDs were commonly not discussed as part of standard care, despite two‐thirds of consumers having discussed ACP with loved ones and approximately half wanting ACP discussions with their doctor.^15^ Ideally, outcomes of ACP discussions will be documented in an advance care directive (ACD).^9^ Definitions, terminology and legislative frameworks surrounding ACDs vary internationally.

Broadly, ACDs are voluntary person‐completed documentation of medical treatment preferences and may include future consent to or refusal of health care and/or the appointment of a substitute decision maker (SDM).^10^, ^12^ In Australia, an ACD has been defined as a written document recognized by specific legislation or common law that is completed and signed by a competent adult.^16^, ^17^ Three main types of ACDs are currently used in Australia: (1) state‐specific statutory documents recording preferences for care, (2) state‐specific statutory documents appointing an SDM and (3) non‐statutory documents recognized under common law (these can record preferences for care but not appoint an SDM).^17^, ^18^, ^19^ Sometimes ACP discussions lead to documentation being created on behalf of a person by health professionals, family members or SDMs to guide future medical treatment decision making in the absence of an ACD, but are not provided for within legislation in Australia. For this study, we use the term ‘ACP documentation’ as a catch‐all term for all ACD types created by the person (including statutory and non‐statutory ACDs) and any other non‐ACD documents created on behalf of the person by a health professional or someone else. Non‐ACD documents refer to any formal medically driven documents, usually completed by doctors, that outline treatment plans in the event of deterioration (eg medical orders or Goals of Care) or ACP discussion records produced by a health professional or someone other than the person.

ACP documentation must be available where the person is receiving care to inform medical decision making.^20^, ^21^ Mechanisms exist in some health services to support people to complete ACDs or elicit existing documentation upon admission to hospital or residential aged care facilities (RACFs, ie long‐term care facility for older adults who can no longer live at home). Health services in Australia use a range of systems to record, store and retrieve ACP documentation, including electronic medical records, hard copy files and the centralized national e‐health system, 'My Health Record'.^22^, ^23^ When these documents are not available, the person may receive care that is inconsistent with their expressed preferences which may be a health‐care safety and quality issue.

ACDs and the requirement for health professionals to act per a person's documented preferences are supported by legislation and national policy frameworks in Australia.^16^, ^17^, ^18^, ^24^, ^25^, ^26^ However, the availability and perceived validity of ACDs are reported as barriers to ACD adherence.^27^, ^28^ Nationally and internationally, ACD uptake remains low, with reported prevalence rates ranging from less than 1% to approximately 30%.^29^, ^30^, ^31^, ^32^, ^33^, ^34^, ^35^ Where ACDs are found, barriers to health practitioner adherence to ACDs include quality concerns such as document currency (eg age of document) and confusion over the legal standing of ACP documentation.^27^, ^28^

The prevalence of ACDs versus non‐ACD documents completed by a health professional or someone else may indicate how well Australians are being supported to complete and store person‐driven documentation. To the best of the research group's knowledge, no Australian study has described ACP uptake across health sectors and jurisdictions by examining the uptake and quality of ACDs as a proportion of ACP documentation, person‐level predictors associated with producing an ACD or the quality of ACDs present. A 2017 Australian multi‐centre pilot prevalence feasibility study^36^, ^37^ found 30% (unweighted) of older people had at least one ACD in their health record, and 22% (unweighted) of people had ACP documentation other than ACDs in their health records, including documents written by health professionals or family members. It is critical to continue to build upon our current knowledge of ACP documentation, ACD prevalence [36, 37] and predictors and quality of ACDs to identify ACP improvement priorities for the Australian health system.

This study aimed to:

1. Describe the prevalence of all types of ACP documentation among older people accessing GPs, hospitals and RACFs, including the proportion and type categorized as ACD and non‐ACD documents
2. Determine person‐level predictors associated with ACD completion, and
3. Identify the quality of ACDs as determined by their alignment with quality indicators (eg personal information, signatories, witnessing requirements and date of completion).

​

## 2 METHODS

This article reports major findings from a national multi‐centre cross‐sector audit study examining the prevalence of ACP documentation, and person‐level characteristics and quality of ACDs in selected Australian general practices (GPs), hospitals and RACFs. The protocol has been published elsewhere.^38^ Learnings from the pilot feasibility study^37^ have informed modifications to study design, site recruitment processes, data items, collection and tools, and data collector training. Ethics approval was obtained from Austin Health Human Research Ethics Committee, Melbourne, Australia (ref: HREC/18/Austin/109), and site‐specific approvals were obtained where required. Findings are reported according to the Strengthening the Reporting of Observational Studies in Epidemiology (STROBE) guidelines.^39^

### 2.1 Setting, sample, recruitment and data collection

Participating sites were GPs, hospitals and RACFs in Australia, recruited via an expression of interest process advertised through key stakeholder networks. Following initial recruitment, additional organizations were approached by the project team to promote representativeness across sectors and jurisdictions.^38^ Organizations were eligible to participate if they had (1) access to a minimum of 30 health records likely to meet inclusion criteria for the audit and (2) sufficient staff and resources to conduct the study. Independent data collectors were provided as needed to undertake the audit at sites that were otherwise unable to participate due to limited resources.

Up to three data collectors for each site completed mandatory online training and had access to state‐specific data collection manuals (reflecting jurisdictional differences in ACP legislation, documentation and terminology). Data collectors were provided with clear definitions and examples for document classification and flowcharts to assist in identifying and classifying documents.^38^ Participating sites were required to nominate how many records they would audit in advance (minimum of 30, maximum of 50). No funding was provided to participating sites.

For health records to be eligible for the audit, patients/residents needed to be aged 65 years or older and admitted to a participating hospital or RACF for more than 48 hours before the audit, or visiting a participating GP on the study day(s). Records were randomly selected from a list of eligible people in hospitals and RACFs, while consecutive eligible records were audited in GPs.^38^

Data collectors searched each record for a maximum of 15 minutes. This timeframe was applied to reflect the need for ACP documentation to be quickly and easily accessible in clinical settings. Information about any ACP documentation identified in the record was extracted (eg type of document, time taken to find, characteristics), together with demographic and clinical information about the person. All data were entered and stored on a secure online purpose‐built database, which was adapted from the pilot feasibility study based on key learnings.^37^, ^38^

### 2.2 Health record audit

Demographic and clinical data extracted included age; gender; country of birth; English language status; relationship status; current/active medical condition(s); palliative care status; and functional status. Functional status was rated using the Eastern Cooperative Oncology Group (ECOG)^40^ status (0 = *Fully active* to 4 = *Completely disabled*) if available, or by estimation based on information in the record if ECOG was not available.

Document characteristics extracted included the presence and type of (1) ACDs completed by the person, (2) non‐ACD documents completed by a health professional and (3) non‐ACD documents completed by someone else (eg family, carer, SDM). For this study and consistent with Australian law, ACDs were defined as documents recognized by statutory legislation (statutory ACD) or common law (non‐statutory ACD) that are completed and signed by a competent adult.^17^ Data collectors were trained to categorize ACDs as *statutory ACD preferences for care*, *statutory ACD‐SDM* or *non‐statutory ACD*. Non‐ACD documents completed by a health professional were categorized as either (1) a medical order (medically driven documents, usually completed by doctors, which outline treatment plans in the event of deterioration) or (2) ACP discussion record.

Quality indicators as determined by its alignment with legislative formalities and the National Framework for ACDs^18^ were collected for ACDs only. These criteria included the presence of patient identification data including their full name, date of birth and address, the presence of the person's signature, the date the document was signed and/or produced and witnessing of the document by a legally appropriate witness. No data related to quality indicators were collected for other ACP documentation, given there is no provision within legislation for non‐ACD documents, and the extensive variability across these documents prevents reasonable comparisons of document quality.

After collecting data, analysis of text descriptions of the names of identified ACDs indicated that 15 documents (1.4% of all ACDs) were misclassified by data collectors as statutory ACD preferences for care and were recoded before analysis as statutory ACD‐SDM (n = 2), non‐statutory ACDs (n = 11) and medical orders (n = 2).

### 2.3 Outcome measures

The primary outcome was ACP documentation prevalence by setting and type, including the prevalence of ACDs by document type, non‐ACD documents completed by a health professional and non‐ACD documents completed by someone else (eg family, SDM). Secondary outcomes were person‐level predictors of ACDs and document quality of ACDs.

### 2.4 Statistical analysis

Statistical analyses were conducted using SPSS v24.0 (IBM). Prevalence rates of different types of ACP documentation were calculated by health sector and overall.

Given disproportionately high numbers of people in the ‘old‐old’ age ranges (ie those aged 75 years and above), and people from particular jurisdictions, a weighting score was derived for each record, in order to calculate a weighted estimate of ACP documentation prevalence among the underlying population of Australians aged 65 years and over. Weighting scores were derived using Australian Bureau of Statistics population data for age, gender and state.^41^ Due to lower response rates in three states/territories (Australian Capital Territory, Tasmania and Western Australia), participants from these states/territories were combined in the weighting process. Population values are not available for gender other than male and female. Where gender was coded as ‘other’, the average weights for males and females of the same age and jurisdiction were applied. The formula for generating weights and the table of derived weights is provided in Supplementary File, Table 1. The relatively over‐represented sub‐groups (by age, gender and state) were used as baseline values in weighted analyses.

Multivariate logistic regression was used to assess the relationship between person‐level predictors and the presence of an ACD completed by the person. A pseudo intra‐class correlation coefficient was calculated to estimate the influence of clustering of observations by site, and a random intercept regression model with independent covariance structure was fitted, to account for this clustering. For each predictor, adjusted and unadjusted results are provided. These model results also include adjustment based on the weighting scores discussed above. In the adjusted model, 876 participants (20.9%) were excluded from the regression analysis due to missing data. For some variables, there were little missing data, but notable were country of birth (n = 347), palliative care status (n = 299), relationship status (n = 322) and functional disability (n = 200). A sensitivity analysis was performed, including missing data as a separate level of each variable to assess the influence of excluding missing data on the results. The analysis indicated that the estimated odds ratios were highly consistent between both models. Therefore, missing data were excluded from the final model. Odds ratios for having an ACD are reported, together with 95% confidence intervals (CI). The statistical significance level was set at 0.05.

Multivariable multinomial mixed‐effects logistic regression was used to assess the relationship between demographic characteristics of the sample with the ACP documentation completion outcome. Three groups composed the outcome: ACDs completed by the person, non‐ACD documents completed by another and no ACP documentation. A random intercept with independent covariance structure was used to account for clustering of observations by site. For each predictor, crude or unadjusted and adjusted results have been provided. These model results also include adjustment based on the weighting discussed above.

Data reported include unweighted prevalence rates by document type and sector and overall weighted prevalence rates for document types. Sub‐category prevalence rate percentages are presented as a percentage of health records containing some form of ACP documentation, rather than as a percentage of all audited health records.

### 2.5 Document quality analysis

Quality data extracted from ACDs were evaluated against criteria for quality sourced from ACP legislative formalities, the National Framework for ACDs and an Australian study.^17^, ^18^, ^42^ Yes/no responses were recorded for each ACD to identify the presence (yes) or absence (no) of quality indicators. Yes responses were used to calculate the total percentage of documents containing information aligning with each quality indicator. Data were then organized by ACD type to describe the overall quality of ACDs by document type. Quality indicators assessed included the full name, date of birth, address and signature of the person, witness signature and their relationship to the person or professional role, and the document date. Document age was calculated from document date.

## 3 RESULTS

Data were collected from 100 organizations between October 2018 and January 2019. Sites included 15 GPs, 27 hospitals and 58 RACFs representing all eight Australian jurisdictions. Of 4188 audited records, one person fell outside of specified age criteria and was excluded, leaving 4187 participants. The median age of participants was 82 years, and 60.3% were female (Table 1). Most were born in Australia (64.2%) and spoke English (89.6%). Participants had a median of three medical conditions: the most common being musculoskeletal/ connective tissue (53.7%), heart condition (52.4%) and dementia (33.2%). More than half had severe to very severe disability.

**TABLE 1:** Sample characteristics^a^ (n = 4187)

Characteristic	n (%)
Age (years)	
Median (interquartile range)	82 (14)
Sex	
Female	2525 (60.3)
Male	1647 (39.3)
Other/unknown	15 (0.3)
Sector	
General practice	676 (16.1)
Hospital	1122 (26.8)
Residential aged care facility	2389 (57.1)
Jurisdiction	
Australian Capital Territory	127 (3.0)
New South Wales	1187 (28.3)
Northern Territory	290 (6.9)
Queensland	850 (20.3)
South Australia	420 (10.0)
Tasmania	50 (1.2)
Victoria	1118 (26.7)
Western Australia	145 (3.5)
Country of birth	
Australia	2688 (64.2)
Other	1152 (27.5)
Unknown	347 (8.3)
Language status	
English‐speaking	3750 (89.6)
Interpreter required	277 (6.6)
Unknown	160 (3.8)
Current relationship status	
Married/de facto	1452 (34.7)
Divorced/separated	387 (9.2)
Widowed	1612 (38.5)
Single	408 (9.7)
Unknown	328 (7.8)
Medical condition(s)^b^	
Cancer (malignant)	628 (15.0)
Dementia	1388 (33.2)
Heart condition	2194 (52.4)
Respiratory condition	998 (23.8)
Chronic kidney condition	445 (10.6)
Endocrine/metabolic/nutritional	1343 (32.1)
Gastrointestinal condition	1043 (24.9)
Neurological condition	959 (22.9)
Urinary or reproductive condition	884 (21.1)
Mental health condition	1370 (32.9)
Musculoskeletal/connective tissue	2250 (53.7)
Other	516 (12.3)
Number of current medical conditions	
0	73 (1.7)
1	608 (14.5)
2	766 (18.3)
3 or more	2740 (65.4)
Median (interquartile range)	3 (2)
Receiving palliative care	
Yes	327 (7.8)
No	3561 (85.0)
Unknown	299 (7.1)
Functional status^c^	
No disability	440 (10.5)
Some disability	451 (10.8)
Moderate disability	914 (21.8)
Severe disability	1506 (36.0)
Very severe disability	676 (16.1)
Insufficient information to determine	200 (4.8)

^a^ Reported as median and interquartile range for continuous variables and as frequency (percentage) for categorical variables.

^b^ Participant may have more than one medical condition.

^c^ Assessed using ECOG performance status, where available, and estimated based on information in the health record where ECOG not available.

### 3.1 Prevalence of all types of advance care planning documentation

Less than half the sample (46.5%, n = 1946) had at least one type of ACP documentation present in their health record. After weighting, the prevalence of ACP documentation was 29.2%. Of the entire sample, 808 records (19.3% unweighted, 10.7% weighted) contained only an ACD, 245 (5.9% unweighted, 6.5% weighted) contained only non‐ACD documents completed by a health professional only, and 556 (13.3% unweighted, 7.2% weighted) contained only non‐ACD documents completed by someone else. Just over a fifth of all records (n = 885, 21.1% unweighted, 15.0% weighted) contained only non‐ACD documents completed by a health professional and/or someone else (ie did not also have a person‐completed document).

### 3.2 Prevalence of advance care directives

The overall (unweighted) prevalence of having at least one ACD was 25.3% (n = 1061). After weighting, the prevalence of having at least one ACD was 14.2%. *Statutory ACD‐SDM* were the most frequently identified ACD document type (12.2% unweighted, 6.1% weighted, n = 511) followed by *non‐statutory ACDs* (11.5% unweighted, 6.9% weighted, n = 480) and *statutory ACD preferences for care* (5.9% unweighted, 3.6% weighted, n = 249), see Table 2. Health records in RACFs were significantly more likely to contain at least one ACD (37.7% unweighted) than those in hospitals (11.1% unweighted) and GPs (5.5% unweighted), *χ* ^2^ (2) = 454.16, *φ* = 0.33, *P* < .001.

**TABLE 2:** Prevalence and types of ACP documentation completed by the person, by a health‐care professional and/or by someone else (family, carer, etc) by health sector and overall

Type of documentation	Unweighted				Weighted
	GP (n = 676) n (%)	Hospital (n = 1122) n (%)	RACF (n = 2389) n (%)	Overall (n = 4187) n (%)	Overall estimate n (%)
No ACP documentation identified	602 (89.1%)	892 (79.5%)	747 (31.3%)	2241 (53.5%)	70.8%
ACD by the person^a^	37 (5.5%)	124 (11.1%)	900 (37.7%)	1061 (25.3%)	14.2%
Statutory ACD preferences for care^a^	6 (0.9%)	36 (3.2%)	207 (8.7%)	249 (5.9%)	3.6%
Statutory ACD‐SDM^a^	22 (3.3%)	93 (8.3%)	396 (16.6%)	511 (12.2%)	6.1%
Non‐statutory ACD^a^	21 (3.1%)	30 (2.7%)	429 (18.0%)	480 (11.5%)	6.9%
ACD by the person only^b^	30 (4.4%)	110 (9.8%)	668 (28%)	808 (19.3%)	10.7%
Non‐ACD documents completed by a health‐care professional^a^	42 (6.2%)	606 (54.0%)	856 (35.8%)	1504 (35.9%)	9.7%
Medical order^a^	1 (0.1%)	545 (48.6%)	599 (25.1%)	1145 (27.3%)	‐
ACP discussion records^a^	42 (6.2%)	95 (8.5%)	354 (14.8%)	491 (11.7%)	‐
Non‐ACD documents completed by a health‐care professional only^b^	35 (5.2%)	87 (7.2%)	129 (5.4%)	245 (5.9%)	6.5%
Non‐ACD documents completed by someone else^a^	2 (0.3%)	29 (2.6%)	726 (30.4%)	757 (18.1%)	10.6%
Non‐ACD documents completed by someone else only^b^	2 (0.3%)	21 (1.9%)	533 (22.3)	556 (13.3%)	7.2%

Note

Abbreviations: ACP, advance care planning; ACD, advance care directive; SDM, substitute decision maker; GP, general practice; RACF, residential aged care facility.

^a^ Totals may not be equal as more than one can apply.

^b^ Refers to those health records containing only one document.

### 3.3 Prevalence of non‐advance care directive documents completed by a health professional

The overall (unweighted) prevalence of non‐ACD documents completed by a health professional was 35.9% (weighted prevalence 9.7%, n = 1504), see Table 2. Roughly one fifth (21.3% unweighted, 6.5% weighted, n = 891) of the audited records only contained non‐ACD documents completed by a health professional (ie did not also have a person‐completed ACD or ACP documentation by someone else). By health‐care sector, prevalence of health professional completed documentation was higher in hospitals (54.0%, n = 606) and RACFs (35.8%, n = 856) than GPs (6.2%, n = 42). Medical orders (27.3% n = 1145) were more prevalent than other types of ACP discussion records produced by a health professional (11.7%, n = 491). By health‐care sector, medical orders were more common in hospitals (48.6%, n = 545) than RACFs (25.1%, n = 599) and GPs (0.1%, n = 1), while other types of ACP discussion records were more common in RACFs (14.8%, n = 354) than hospitals (8.5%, n = 95) and GPs (6.2%, n = 42). Other types of ACP discussion records completed by a health professional included ACP clinical notes and letters.

### 3.4 Prevalence of non‐advance care directive documents completed by someone else

The prevalence of non‐ACD documents completed by someone else was 18.1% (weighted prevalence 10.6%, n = 757), see Table 2. Prevalence was much higher in RACFs (30.4%, n = 726) than hospitals (2.6%, n = 29) and GPs (0.3%, n = 2). 10.9% of the audited records contained only ACP documentation completed by someone else (n = 455, weighted prevalence 7.2%).

### 3.5 Prevalence of advance care directives as a proportion of all ACP documentation

ACDs comprised 25.3% (n = 1061, 14.2% weighted) of all health records audited (n = 4187, see Table 2). Statutory ACD preferences for care accounted for 5.9% (n* = *249, 3.6% weighted), statutory ACD‐SDMs accounted for an additional 12.2% (n* = 511*, 6.1% weighted), and non‐statutory ACDs accounted for 11.5% (n* = 480*, 6.9% weighted) of all ACP documentation. Non‐statutory ACDs were most prevalent in RACFs (n = 429, 18.0% unweighted) as compared to GPs and hospitals.

### 3.6 Predictors of advance care directives completed by the person

Adjusted and unadjusted results of the measured predictors of having an ACD completed by the person (versus no ACP documentation or some other ACP discussion record) are provided in Table 3. The site effect was estimated to be 1.21 (pseudo ICC value of 0.269), indicating that responses within sites were correlated, with an overall strong effect of site on the results. Across the sample, the adjusted model revealed that the odds of having an ACD (vs not having an ACD) were higher for those who were female (compared with male), older, divorced/separated (compared to those who were widowed), had two or more medical conditions (compared with one medical condition), receiving palliative care (compared with not receiving palliative care) and/or residing in a RACF (compared with being in a hospital or general practice).

**TABLE 3:** Multivariate logistic regressions predicting the presence of an advance care directive completed by the person^a^

Variable	Baseline	Reference	Unadjusted OR (95% CI)	*P*‐value	Adjusted OR (95%CI)	*P*‐value
Gender	Male	Female	1.46 (1.18, 1.79)	<.001***	1.29 (1.01, 1.66)	.042*
	Male	Other/unknown	1.79 (0.31, 10.4)	.519	4.11 (0.37, 45.8)	.250
	Female	Other/unknown	1.23 (0.21, 7.13)	.820	3.17 (0.29, 35.7)	.346
Age (years)			1.05 (1.03, 1.07)	<.001***	1.04 (1.02, 1.06)	<.001***
Jurisdiction	New South Wales	Australian Capital Territory	0.33 (0.05, 2.35)	.270	0.78 (0.18, 3.42)	.121
	New South Wales	Northern Territory	0.27 (0.03, 2.41)	.242	4.30 (0.17, 110)	.228
	New South Wales	Queensland	1.29 (0.50, 3.31)	.594	1.37 (0.66, 2.85)	.377
	New South Wales	South Australia	1.51 (0.44, 5.14)	.513	1.79 (0.69, 4.63)	.392
	New South Wales	Tasmania	0.86 (0.04, 19.9)	.924	NA	NA
	New South Wales	Victoria	1.03 (0.41, 2.59)	.955	1.25 (0.60, 2.62)	.739
	New South Wales	Western Australia	0.15 (0.03, 0.94)	.042*	0.33 (0.08, 1.35)	.961
Multimorbidity	3+ medical conditions	1 medical condition	0.18 (0.12, 0.29)	<.001***	0.27 (0.15, 0.48)	<.001***
	3+ medical conditions	2 medical conditions	0.64 (0.49, 0.85)	<.001***	0.97 (0.70, 1.35)	.875
	1 medical condition	2 medical conditions	3.51 (2.15, 5.73)	<.001***	3.62 (1.95, 6.72)	<.001***
Country of birth	Australia	Other	1.08 (0.83, 1.41)	.558	1.07 (0.80, 1.43)	.644
Receiving palliative care	Yes	No	0.47 (0.32, 0.69)	<.001***	0.50 (0.33, 0.76)	.001**
Relationship status	In a relationship	Single	0.99 (0.72, 1.35)	.940	0.84 (0.58, 1.22)	.356
	In a relationship	Divorced/separated	1.26 (0.91, 1.72)	.161	1.24 (0.88, 1.77)	.211
	In a relationship	Widowed	1.17 (0.88, 1.56)	.280	0.83 (0.60, 1.14)	.249
	Single	Divorced/separated	1.27 (0.88, 1.84)	.203	1.49 (0.99, 2.24)	.055
	Single	Widowed	1.19 (0.84, 1.67)	.332	0.99 (0.65, 1.48)	.943
	Divorced/separated	Widowed	0.93 (0.66, 1.33)	.702	0.66 (0.45, 0.97)	.035*
Functional disability	None/some	Moderate	1.52 (1.04, 2.22)	.032*	1.10 (0.69, 1.75)	.687
	None/some	Severe/very severe	2.00 (1.38, 2.88)	<.001***	1.10 (0.71, 1.69)	.679
	Moderate	Severe/very severe	1.31 (0.85, 1.76)	.064	1.00 (0.73, 1.37)	.981
Sector	RACF	General practice	0.09 (0.04, 0.20)	<.001***	0.12 (0.03, 0.45)	.002**
	RACF	Hospital	0.12 (0.60, 0.21)	<.001***	0.18 (0.09, 0.36)	<.001***
	General practice	Hospital	1.34 (0.52, 3.42)	.543	1.53 (0.39, 6.02)	.543

Note

Abbreviations: CI, confidence interval; OR, odds ratio; RACF, residential aged care facility.

^a^ Only ACDs completed by the person were considered in this analysis (not other types of ACP documentation).

* Significant at *P* = .05,

** Significant at *P* = .01,

*** Significant at *P* < .001.

### 3.7 Quality of advance care directives

All three types of ACDs included in the quality assessment contained some records that were missing one or more quality indicators (Tables 4 and 5). Most documents contained the person's full name (n = 1145, 92.3%), their date of birth (n = 703, 56.7%), the person's address (n = 989, 79.8%), were signed by the person (n = 1113, 89.8%), were witnessed (n = 1106, 89.2%) and dated (n = 1181, 96.6%). Overall, more than half (n = 722, 58.2%) of ACDs were three or more years old, and less than a quarter (n = 274, 22.1%) were created within 12 months of the audit date.

**TABLE 4:** Quality of advance care directives (n = 1061)^a^

Characteristic	Presence	Statutory ACD: preferences for care (n = 249)	Statutory ACD: SDM (n = 511)	Non‐statutory ACD (n = 480)	Total (n = 1240)
		n (%)	n (%)	n (%)	n (%)
Contains person's full name	Yes	239 (96.0)	501 (98.0)	405 (84.4)	1145 (92.3)
	No, only part of their name	9 (3.6)	8 (1.6)	45 (9.4)	62 (5.0)
	No, name not present	1 (0.4)	0 (0.0)	0 (0.0)	1 (0.1)
	Missing data	0 (0.0)	2 (0.4)	30 (6.2)	32 (2.6)
Contains person's date of birth	Yes	222 (89.2)	135 (26.4)	346 (72.1)	703 (56.7)
	No	27 (10.8)	374 (73.2)	104 (21.7)	505 (40.7)
	Missing data	0 (0.0)	2 (0.4)	30 (6.2)	32 (2.6)
Contains person's address	Yes	194 (77.9)	482 (94.3)	313 (65.2)	989 (79.8)
	No	55 (22.1)	27 (5.3)	137 (28.6)	219 (17.7)
	Missing data	0 (0.0)	2 (0.4)	30 (6.2)	32 (2.6)
Signed by the person	Yes	237 (95.2)	494 (96.7)	382 (79.6)	1113 (89.8)
	No^b^	12 (4.8)	15 (2.9)	68 (14.2)	95 (7.7)
	Missing data	0 (0.0)	2 (0.4)	30 (6.2)	32 (2.6)
Witnessed	Yes	235 (94.4)	497 (97.3)	374 (77.9)	1106 (89.2)
	No	14 (5.6)	12 (2.3)	73 (15.2)	99 (8.0)
	Missing data	0 (0.0)	2 (0.4)	33 (6.9)	35 (2.8)
Witnessed/signed by^c^	Doctor	88 (35.3)	34 (6.7)	185 (38.5)	307 (12.3)
	Other health‐care professional	23 (9.2)	33 (6.5)	120 (25.0)	176 (7.0)
	Legal practitioner	48 (19.3)	300 (58.7)	13 (2.7)	361 (14.5)
	Justice of the Peace/public notary	112 (45.0)	125 (24.5)	7 (1.5)	244 (9.8)
	Substitute decision maker	24 (9.6)	5 (1.0)	128 (26.7)	157 (6.3)
	Someone else	235 (94.4)	497 (97.3)	374 (77.9)	1106 (44.3)
	Unable to determine witness	11 (4.4)	25 (4.9)	42 (8.8)	78 (3.1)
	Other	17 (6.8)	12 (2.3)	39 (8.1)	68 (2.7)
Dated	Yes	240 (96.4)	505 (98.8)	434 (90.4)	1179 (95.1)
	No	9 (3.6)	4 (0.8)	18 (3.8)	31 (2.5)
	Missing data	0 (0.0)	2 (0.4)	28 (5.8)	30 (2.4)
Age of document^d^	Less than 12 mo	54 (21.7)	58 (11.3)	162 (33.7)	274 (22.1)
	1‐2 y	37 (14.9)	52 (10.2)	91 (19.0)	180 (14.5)
	3‐5 y	77 (30.9)	145 (28.4)	131 (27.3)	353 (28.5)
	More than 5 y	70 (28.1)	249 (48.7)	50 (10.4)	369 (29.8)
	Missing data	11 (4.4)	7 (1.4)	46 (9.6)	64 (5.2)

^a^ Participants (n = 1061) may have had more than one type of document.

^b^ No may include those that cannot physically sign.

^c^ More than one signatory may have been recorded, total signatories n = 2497.

^d^ Age at the time of the study. ACD, advance care directive.

**TABLE 5:** Presence of preferred patient identifiers within advance care directives

	Patient identifiers present in document	Statutory ACD: preferences (n = 249)		Statutory ACD: SDM^a^ (n = 511)		Structured non‐statutory ACD (n = 463)		All ACDs (n = 1223)	
		n	*%*	n	*%*	n	*%*	n	*%*
6 patient identifiers	Name, DOB, Signature, Dated, Witnessed & Address	159	63.9%	62	12.1%	103	22.2%	324	26.5%
5 patient identifiers	Name, DOB, Signature, Dated & Witnessed	157	63.1%	61	11.9%	102	22.0%	320	26.2%
	Name, DOB, Signature, Dated & Address	199	79.9%	81	15.9%	164	35.4%	444	36.3%
	Name, DOB, Signature, Witnessed & Address	167	67.1%	62	12.1%	196	42.3%	425	34.8%
	Name, DOB, Dated, Witnessed & Address	159	63.9%	62	12.1%	103	22.2%	324	26.5%
	Name, Signature, Dated, Witnessed & Address	163	65.5%	62	12.1%	120	25.9%	345	28.2%
	DOB, Signature, Dated, Witnessed & Address	175	70.3%	309	60.5%	125	27.0%	609	49.8%
4 patient identifiers	Name, DOB, Signature & Dated	209	83.9%	129	25.2%	277	59.8%	615	50.3%
	Name, DOB, Signature & Witnessed	199	79.9%	128	25.0%	164	35.4%	491	40.1%
	Name, DOB, Signature & Address	167	67.1%	109	21.3%	196	42.3%	472	38.6%
	Name, Signature, Dated & Witnessed	219	88.0%	482	94.3%	196	42.3%	897	73.3%
	Name, Signature, Dated & Address	183	73.5%	465	91.0%	252	54.4%	900	73.6%
	Name, Signature, Witnessed & Address	175	70.3%	460	90.0%	125	27.0%	760	62.1%
	Name, Dated, Witnessed & Address	180	72.3%	466	91.2%	143	30.9%	789	64.5%
	DOB, Signature, Dated & Witnessed	202	81.1%	130	25.4%	177	38.2%	509	41.6%
	DOB, Signature, Dated & Address	168	67.5%	110	21.5%	206	44.5%	484	39.6%
	DOB, Signature, Witnessed & Address	160	64.3%	110	21.5%	110	23.8%	380	31.1%
	DOB, Dated, Witnessed & Address	165	66.3%	114	22.3%	130	28.1%	409	33.4%
	Signature, Dated, Witnessed & Address	177	71.1%	465	91.0%	135	29.2%	777	63.5%

^a^ Includes ACT and QLD statutory ACD:SDM documents that do not include the date of birth (DOB) field.

Overall, the patient identifier most frequently absent was the person's date of birth (n = 704, 57.6%). By document type, date of birth was most likely to be absent for statutory ACD‐SDM documents (n = 135, 26.4%), while statutory ACD: preferences for care and structured non‐statutory ACDs were least likely to contain the person's address (n = 194, 77.9% and n = 315, 67.8%, respectively). All prescribed ACD forms contained a designated section for date of birth except for statutory ACD‐SDM documents in the Australian Capital Territory (ACT) and Queensland (QLD).

Only 26.5% of all ACDs (n = 324) included the full name, date of birth, address, the signature of the person, document date and the signature of a witness. The document type most likely to contain all six identifiers was statutory ACD: preference for care documents (n = 159, 63.9%), with just 22.2% of non‐statutory ACDs (n = 103) and 12.1% of all statutory ACD: SDM documents (n = 62) containing all six patient identifiers. After removing statutory ACD: SDM documents produced in the ACT or QLD, all six patient identifiers were present in 17.2% statutory ACD: SDM documents (n = 61). These data are available in a Supplementary Table 2.

Just under three‐quarters of all ACDs included the full name, signature, document date and witness (n = 897, 73.3%). This combination of identifiers was found most often in statutory ACD: SDM documents (n = 482, 94.3%), with 88.0% of all statutory ACD: preference for care documents (n = 219) and 42.3% of all structured non‐statutory ACDs (n = 196) including this combination of patient identifiers.

## 4 DISCUSSION

This multi‐centre, cross‐sector audit of the health records of older people accessing Australian health and residential aged care services provides new evidence regarding low prevalence rates (both unweighted and weighted) of ACP documentation across all health‐care sectors. To the best of our knowledge, this is the first study to report findings regarding ACD prevalence as a proportion within ACP documentation, by type, predictors and quality indicators. Less than half of all audited records contained any type of ACP documentation and only a quarter of records contained a person‐completed ACD. After weighting, the overall ACP documentation prevalence rate was low at 29.2%, with just 14.2% of records containing an ACD and just over a third (weighted 15.0%) of the sample only had ACP documentation completed by a health professional and/or someone else. S*tatutory ACD‐SDM* document types were most common, and prevalence rates were highest in the RACF sector. Hospital health records were most likely to contain non‐ACD documents created by a health professional. Almost a fifth of all ACP documentation were non‐ACDs completed by someone else (10.6% weighted). In terms of quality indicators, ACDs often contained one or more missing patient identifiers, and over half were three or more years old, influencing currency. Just under a third of all ACDs included the person's full name, date of birth, address, signature, document date and signature of a witness, with statutory ACD: preferences for care documents most likely to contain all six person identifiers.

The 25% unweighted ACD prevalence rate and the higher prevalence of ACDs in RACFs reflect the prevalence rates reported in the 2017 pilot feasibility study.^36^ The weighted ACD prevalence rate found in the current study is similar to other Australian research reporting a general community ACD prevalence rate of 12^31^ to 14%.^35^ Internationally, higher ACD prevalence rates have been reported, with one systematic review and meta‐analysis reporting a prevalence of any type of ACD for US adults to be 37%.^43^ A separate Canadian study also showed a higher ACD prevalence rate, with 20% of participants reporting having completed an ACD and 47% reporting having appointed an SDM.^44^ Variation in prevalence rates may reflect differences in recruitment processes, prevalence rate calculation approaches (including population weighting), demographic profiles or differences in the storage of ACDs. However, further exploration of this variation is warranted.

When looking at ACD prevalence by sector, ACDs were more prevalent in RACFs, with GP health records least likely to contain any ACDs. Higher rates of ACP documentation in RACFs may reflect the need to plan for future medical treatment decisions due to cognitive decline, palliative or end‐of‐life care in aged care.^45^ Differences in sector processes for identifying ACP documentation on admission and the increased likelihood that an individual's health information will be stored at their RACF as their primary residence may also contribute to this finding. National and international ACD prevalence rates have produced similar results to those in the current study, with hospital prevalence rates reported between 0% and 35%^11^, ^46^, ^47^, ^48^, ^49^, ^50^ and RACF prevalence rates in Canada reported between 11% and 44%.^45^ For GPs, ACD rates have been reported from 20% to 33%^51^, ^52^ with much lower rates reported in Australian GPs.^53^ Possible explanations for the lower prevalence of ACDs in GPs may include a lack of systems, processes, time and/or funding in place to support ACP discussions with patients or issues with the approach used by general practitioners to identify patients they believe would benefit from ACP discussions.^13^, ^54^, ^55^, ^56^

The low prevalence rates for ACP documentation and especially ACDs across health‐care sectors in this study are concerning given the importance of person‐centred care and needs of a study population with a median age of 82 years, a median of three medical conditions, with more than half showing severe or very severe functional decline, half having heart disease and one‐third having dementia. Many of these people might be expected to have a limited prognosis, be at risk of deteriorating health and require significant medical treatment decisions to be made.

Non‐ACD documents, including medical orders and ACP discussion records, completed by a health professional were more common in hospitals than RACFs and GPs. This result is likely to reflect jurisdictional and hospital policy promoting the regular use of medical orders during patient admissions to hospital. It may also reflect medical practitioner preference to use doctor completed rather than person‐completed documentation, or as a result of sudden deterioration or delirium after admission.^15^ Research suggests health professionals prefer using clinician‐led documents, as these are often easier to translate into clinical plans than general instructions contained in ACDs that may or may not relate to the illness being treated.^27^ Alternatively, the higher prevalence of medical orders in hospitals than ACDs may reflect system‐specific factors and/or processes that limit the amount of time health professionals can spend discussing ACP with patients or a lack of processes to assist with locating an ACD. Given the purpose of ACP is to promote person‐driven planning for future medical treatment decision making, further research is needed to examine whether or not health professionals are encouraging the person receiving care to create an ACD rather than creating non‐ACD documents on behalf of a person.

After weighting to account for the larger proportion of ACP documentation found in RACF health records, the prevalence of non‐ACD documents completed by someone else was 10.6%, with the majority found in RACFs. Ideally, future health‐care planning should result in the earlier completion of one or more person‐driven ACDs. However, some individuals may be unwilling or unable to complete an ACD because of cognitive decline, cultural factors, poor literacy or distrust.^57^, ^58^ In these scenarios, others may decide to create non‐ACD documents on their behalf to inform future care decisions.

Several person‐level predictors were associated with higher odds of having an ACD on record. Women and those who were older were more likely to have an ACD in their health record. These results are consistent with existing research.^30^, ^31^, ^45^, ^46^, ^59^, ^60^, ^61^ Those who were divorced or separated also had significantly higher odds of having an ACD in their health record compared to those who were widowed. This result suggests people may be more likely to complete documentation outlining preferences for care or assigning an SDM if they no longer have a close and continuing relationship with the person who previously would have been called upon to make decisions on their behalf.^62^ Higher prevalence rates for those who are not married or in a de facto relationship further suggest the absence of a significant other may encourage people to complete a statutory ACD appointing an SDM. However, further research is needed to determine whether relationship status has a robust effect on the likelihood of having an ACD on record, as these results are not conclusive. Further research could also explore motivating factors for a person or how treating teams identify priority individuals to create an ACD. For example, the need to undertake ACP when cognitive impairment is foreseeable has been identified as critical, and warrants further investigation.^63^

More than two‐thirds of those with an ACD on file had two or more comorbidities, and just over half had severe or very severe functional impairment. Other research has also identified an association between the presence of an ACD with health status and/or multimorbidity.^45^, ^46^, ^60^ Consistent with previous research, people receiving palliative care were also more likely to have an ACD.^46^, ^59^, ^60^ These associations may reflect the way that health professionals identify individuals they deem appropriate for conversations about ACP, or the greater proportion of people living in RACFs in this study.

The results of the quality assessment suggest ACD prevalence rates may overestimate the presence of documents that are likely to be enacted by a health practitioner at a person's point of care. National policy guidance in Australia recommends an ACD should use the prescribed form when available and contain quality indicators such as name, document date, person signature and witnessing.^17^, ^18^, ^64^, ^65^ The presence of these quality indicators allows health practitioners to verify the identity of the patient the document applies to and provides evidence the document was created voluntarily by the person at a time they had decision‐making capacity.^17^, ^18^ However, only 73% of ACDs included all four of these patient identifiers, and 58% of ACDs were found to be three or more years old. These findings suggest that consumers may require more support to complete quality ACDs. While an ACD generally does not expire unless specified, people who have decision‐making capacity should be encouraged to update documents if their health status and preferences change.

Although the absence of a single quality indicator may not result in health professionals questioning the legitimacy of the document, the absence of specific quality indicators or combinations of quality indicators may cause a health professional to question the document's legitimacy. Research shows health professionals may disregard ACDs if they are concerned the document's quality raises questions about whether the document is a genuine reflection of the person's preference.^28^, ^29^ In instances where doctors question the legitimacy of ACDs, they are likely to apply their own medical decision making, which may or may not align with the individual's treatment preferences.

National and international studies have identified a lack of congruence between patient preferences and medical treatment decision making.^66^, ^67^, ^68^, ^69^ In a study of people with end‐stage kidney disease, 15 of 57 participants received medical treatment that was inconsistent with their known preferences at end of life.^70^ An international study of emergency physicians found 86% of respondents were willing to honour legal ACDs. When informal ACP documentation was used, only 7% were willing to adhere to the specified preferences. ^66^ Similarly, a study of 649 medical specialists found only 32% of specialists complied with the law when presented with an ACD scenario, with doctors more likely to make clinically indicated medical decisions than make medical decisions that follow the law.^68^

When examining ACD quality indicators by document type, considerable differences are apparent, with 64% of *ACD preferences for care,* 12% of *statutory ACD‐SDMs* and 22% of *non‐statutory ACDs* containing the full name, date of birth, address and signature of the person as well as the date of the document and signature of at least one witness. Missing ACD quality indicators may reflect limitations of available ACD forms. Of note, the ACT and QLD guardianship forms do not have a field for date of birth. These findings have implications for the design of the relevant forms to ensure they reflect quality indicators established by the National Framework for ACDs and jurisdictional legislation.^18^

A lack of accessible, person‐completed and quality ACDs represents an important ACP implementation issue for Australian health and aged care services. At an individual level, these factors could have devastating implications on a person's care, affect medical decision making and be considered a medical error.^71^ Beyond the implications for the provision of care that aligns with a person's preferences, this study indicates a better understanding of the systems, organizational and clinician characteristics that affect ACP implementation is required.

### 4.1 Strengths and limitations

The strengths of this study include the cross‐sector involvement, participation across all Australian jurisdictions, comprehensive training of auditors, reliability testing and national research governance. However, this research has several limitations. Recruiting via expression of interest likely resulted in a sample containing primarily services with an interest in the study topic. Weighted analyses contributed to a more representative description of ACP in Australia; however, disproportional results across organization types and jurisdictions still limit the generalizability of findings across Australia. Similarly, weighting was performed for some variables but could not be weighted for others, such as site. Because not all Australian jurisdictions had representation from each sector, caution should be used when interpreting results related to jurisdictions, and the presence of missing data may have also influenced results. Where more than one document was present in a health record, only the date of the most recent document was recorded. As such, documents with no date identified were removed from the quality analysis and listed as missing data. Data collected included assessments of functional status, but not cognitive status. Cognitive status may be a stronger predictor of whether an ACD exists than a person's functional status if this assessment is made at the time an ACD is created. The quality analysis did not consider whether ACD content could be used in a clinical setting to inform decision making. Lastly, it was not possible to report quality data by jurisdiction due to a lack of representation.

## 5 CONCLUSION

Low prevalence of all types of ACP documentation and a lack of accessible, person‐completed and quality ACDs were found in Australian GPs, hospitals and RACFs. After weighting, only 29% of people aged 65 years or older had any ACP documentation, and only 14% had one or more ACDs available in their records at their point of care. Older Australians are more likely to have non‐ACD documents completed by a health professional or someone else. This study suggests that there is limited documented planning for future medical treatment decision making, and the development and storage of a person‐completed ACD is not routinely part of care in Australia. When ACDs are present, their application may be limited by quality issues. There are implications for sectors, services providers, health practitioners and individuals, to ensure access to and support for ACP. ACP implementation strategies will need to address ways to improve the prevalence and quality of ACDs. Further research exploring the impact of different ACP systems, program characteristics, policy, funding, clinician priorities and/or training on ACD prevalence may be useful.

## CONFLICT OF INTEREST

All authors declare that they have no conflict of interest related to this work.

## AUTHORS’ CONTRIBUTIONS

Kimberly Buck (joint first author) contributed to study design; data acquisition, analysis and interpretation; and drafting manuscript. Linda Nolte (joint first author) contributed to study conceptualization; acquisition of funding; research governance; study design; data analysis and interpretation; and drafting and approval of manuscript. Marcus Sellars contributed to study design; data acquisition, analysis and interpretation; and drafting of manuscript. Authors, Craig Sinclair, Ben P White, Helana Kelly, contributed to study design; data analysis planning; interpretation; and drafting of manuscript. Ashley Macleod contributed to data analysis and interpretation; and drafting of manuscript. Karen M Detering contributed to study conceptualization; study design; data analysis and interpretation; and drafting manuscript.

## Supporting information

Table S2

Supplementary Material

## Data Availability

The data that support the findings of this study are available on request from the corresponding author. The data are not publicly available due to privacy or ethical restrictions.
