# Supplementary material for: Advance care directive prevalence among older Australians and associations with person‐level predictors and quality indicators
Source: Health Expect. 2021 May 1;24(4):1312–25. doi: 10.1111/hex.13264 (PMC8369087; doi:10.1111/hex.13264)
Supplement: Supplementary file 1 — Table S2 [file HEX-24-1312-s001.docx]

**Supplementary Table 2.** Presence of preferred patient identifiers within advance care directives, excluding statutory ACD: SDMs produced in ACT and QLD

|  |  | **Statutory ACD: preferences** (*n*=249) | | **Statutory ACD: SDM (excl. ACT&QLD)** (*n*=354) | | **Structured non-statutory ACD** (*n*=463) | | **All ACDs (excl. ACT&QLD)**  (*n*=1066) | |
| --- | --- | --- | --- | --- | --- | --- | --- | --- | --- |
|  |  | *n* | *%* | *n* | *%* | *n* | *%* | ***n*** | ***%*** |
| 6 patient identifiers | Name, DOB, Signature, Dated, Witnessed & Address | 159 | 63.9% | 61 | 17.2% | 103 | 22.2% | **323** | **30.3%** |
| 5 patient identifiers | Name, DOB, Signature, Dated & Witnessed | 157 | 63.1% | 61 | 17.2% | 102 | 22.0% | **320** | **30.0%** |
|  | Name, DOB, Signature, Dated & Address | 199 | 79.9% | 81 | 22.9% | 164 | 35.4% | **444** | **41.7%** |
|  | Name, DOB, Signature, Witnessed & Address | 167 | 67.1% | 62 | 17.5% | 196 | 42.3% | **425** | **39.9%** |
|  | Name, DOB, Dated, Witnessed & Address | 159 | 63.9% | 62 | 17.5% | 103 | 22.2% | **324** | **30.4%** |
|  | Name, Signature, Dated, Witnessed & Address | 163 | 65.5% | 62 | 17.5% | 120 | 25.9% | **345** | **32.4%** |
|  | DOB, Signature, Dated, Witnessed & Address | 175 | 70.3% | 309 | 87.3% | 125 | 27.0% | **609** | **57.1%** |
| 4 patient identifiers | Name, DOB, Signature & Dated | 209 | 83.9% | 82 | 23.2% | 277 | 59.8% | **568** | **53.3%** |
|  | Name, DOB, Signature & Witnessed | 199 | 79.9% | 81 | 22.9% | 164 | 35.4% | **444** | **41.7%** |
|  | Name, DOB, Signature & Address | 167 | 67.1% | 62 | 17.5% | 196 | 42.3% | **425** | **39.9%** |
|  | Name, Signature, Dated & Witnessed | 219 | 88.0% | 331 | 93.5% | 196 | 42.3% | **746** | **70.0%** |
|  | Name, Signature, Dated & Address | 183 | 73.5% | 313 | 88.4% | 252 | 54.4% | **748** | **70.2%** |
|  | Name, Signature, Witnessed & Address | 175 | 70.3% | 309 | 87.3% | 125 | 27.0% | **609** | **57.1%** |
|  | Name, Dated, Witnessed & Address | 180 | 72.3% | 312 | 88.1% | 143 | 30.9% | **635** | **59.6%** |
|  | DOB, Signature, Dated & Witnessed | 202 | 81.1% | 83 | 23.4% | 177 | 38.2% | **462** | **43.3%** |
|  | DOB, Signature, Dated & Address | 168 | 67.5% | 63 | 17.8% | 206 | 44.5% | **437** | **41.0%** |
|  | DOB, Signature, Witnessed & Address | 160 | 64.3% | 63 | 17.8% | 110 | 23.8% | **333** | **31.2%** |
|  | DOB, Dated, Witnessed & Address | 165 | 66.3% | 64 | 18.1% | 130 | 28.1% | **359** | **33.7%** |
|  | Signature, Dated, Witnessed & Address | 177 | 71.1% | 313 | 88.4% | 135 | 29.2% | **625** | **58.6%** |
