# Supplementary material for: Advance care directive prevalence among older Australians and associations with person‐level predictors and quality indicators
Source: Health Expect. 2021 May 1;24(4):1312–25. doi: 10.1111/hex.13264 (PMC8369087; doi:10.1111/hex.13264)
Supplement: Supplementary file 2 — Supplementary Material [file HEX-24-1312-s002.docx]

**Supplementary Table 1.** Sample representativeness along with derived weights

| **Gender** | **State** | **Age** | **Population** | **Sample** | **Weight** |
| --- | --- | --- | --- | --- | --- |
| female | ACT/TAS/WA | 65-69 | **85147 (2.2%)** | **19 (0.5%)** | 12.25 |
| female | ACT/TAS/WA | 70-74 | **65713 (1.7%)** | **30 (0.7%)** | 3.79 |
| female | ACT/TAS/WA | 75-79 | **47905 (1.3%)** | **39 (0.9%)** | 1.64 |
| female | ACT/TAS/WA | 80-84 | **34149 (0.9%)** | **41 (1.0%)** | 1.06 |
| female | ACT/TAS/WA | 85 and over | **38140 (1.0%)** | **79 (1.9%)** | 0.32 |
| male | ACT/TAS/WA | 65-69 | **83198 (2.2%)** | **23 (0.6%)** | 8.17 |
| male | ACT/TAS/WA | 70-74 | **64152 (1.7%)** | **29 (0.7%)** | 3.96 |
| male | ACT/TAS/WA | 75-79 | **43461 (1.1%)** | **18 (0.4%)** | 6.97 |
| male | ACT/TAS/WA | 80-84 | **27373 (0.7%)** | **15 (0.4%)** | 6.32 |
| male | ACT/TAS/WA | 85 and over | **23049 (0.6%)** | **27 (0.6%)** | 1.64 |
| female | SA | 65-69 | **48669 (1.3%)** | **27 (0.6%)** | 3.47 |
| female | SA | 70-74 | **40217 (1.1%)** | **18 (0.4%)** | 6.45 |
| female | SA | 75-79 | **29366 (0.8%)** | **39 (0.9%)** | 1.00 |
| female | SA | 80-84 | **21792 (0.6%)** | **37 (0.9%)** | 0.83 |
| female | SA | 85 and over | **27936 (0.7%)** | **145 (3.5%)** | 0.07 |
| male | SA | 65-69 | **46069 (1.2%)** | **12 (0.3%)** | 16.62 |
| male | SA | 70-74 | **37461 (1.0%)** | **19 (0.5%)** | 5.39 |
| male | SA | 75-79 | **25592 (0.7%)** | **28 (0.7%)** | 1.70 |
| male | SA | 80-84 | **17642 (0.5%)** | **32 (0.8%)** | 0.89 |
| male | SA | 85 and over | **16532 (0.4%)** | **62 (1.5%)** | 0.22 |
| female | NT | 65-69 | **3594 (0.1%)** | **46 (1.1%)** | 0.09 |
| female | NT | 70-74 | **2159 (0.1%)** | **29 (0.7%)** | 0.13 |
| female | NT | 75-79 | **1312 (0%)** | **25 (0.6%)** | 0.11 |
| female | NT | 80-84 | **734 (0%)** | **11 (0.3%)** | 0.32 |
| female | NT | 85 and over | **601 (0%)** | **12 (0.3%)** | 0.22 |
| male | NT | 65-69 | **4248 (0.1%)** | **61 (1.5%)** | 0.06 |
| male | NT | 70-74 | **2615 (0.1%)** | **43 (1%)** | 0.07 |
| male | NT | 75-79 | **1520 (0%)** | **28 (0.7%)** | 0.10 |
| male | NT | 80-84 | **603 (0%)** | **20 (0.5%)** | 0.08 |
| male | NT | 85 and over | **423 (0%)** | **14 (0.3%)** | 0.11 |
| female | NSW | 65-69 | **195245 (5.2%)** | **33 (0.8%)** | 9.31 |
| female | NSW | 70-74 | **159901 (4.2%)** | **58 (1.4%)** | 2.47 |
| female | NSW | 75-79 | **117754 (3.1%)** | **76 (1.8%)** | 1.06 |
| female | NSW | 80-84 | **87478 (2.3%)** | **135 (3.2%)** | 0.25 |
| female | NSW | 85 and over | **105770 (2.8%)** | **440 (10.5%)** | 0.03 |
| male | NSW | 65-69 | **189261 (5.0%)** | **51 (1.2%)** | 3.78 |
| male | NSW | 70-74 | **154380 (4.1%)** | **56 (1.3%)** | 2.56 |
| male | NSW | 75-79 | **106053 (2.8%)** | **68 (1.6%)** | 1.19 |
| male | NSW | 80-84 | **70658 (1.9%)** | **92 (2.2%)** | 0.43 |
| male | NSW | 85 and over | **63698 (1.7%)** | **173 (4.1%)** | 0.11 |
| female | QLD | 65-69 | **121836 (3.2%)** | **29 (0.7%)** | 7.52 |
| female | QLD | 70-74 | **97789 (2.6%)** | **47 (1.1%)** | 2.30 |
| female | QLD | 75-79 | **67914 (1.8%)** | **64 (1.5%)** | 0.86 |
| female | QLD | 80-84 | **46853 (1.2%)** | **91 (2.2%)** | 0.29 |
| female | QLD | 85 and over | **54159 (1.4%)** | **312 (7.5%)** | 0.03 |
| male | QLD | 65-69 | **120066 (3.2%)** | **41 (1.0%)** | 3.71 |
| male | QLD | 70-74 | **96710 (2.6%)** | **59 (1.4%)** | 1.44 |
| male | QLD | 75-79 | **64037 (1.7%)** | **51 (1.2%)** | 1.28 |
| male | QLD | 80-84 | **39111 (1.0%)** | **58 (1.4%)** | 0.60 |
| male | QLD | 85 and over | **32947 (0.9%)** | **97 (2.3%)** | 0.18 |
| female | VIC | 65-69 | **152083 (4.0%)** | **61 (1.5%)** | 2.12 |
| female | VIC | 70-74 | **122050 (3.2%)** | **79 (1.9%)** | 1.02 |
| female | VIC | 75-79 | **91195 (2.4%)** | **90 (2.2%)** | 0.58 |
| female | VIC | 80-84 | **68048 (1.8%)** | **125 (3%)** | 0.23 |
| female | VIC | 85 and over | **80981 (2.1%)** | **288 (6.9%)** | 0.05 |
| male | VIC | 65-69 | **143259 (3.8%)** | **61 (1.5%)** | 2.00 |
| male | VIC | 70-74 | **115434 (3%)** | **87 (2.1%)** | 0.79 |
| male | VIC | 75-79 | **81108 (2.1%)** | **94 (2.3%)** | 0.48 |
| male | VIC | 80-84 | **54403 (1.4%)** | **92 (2.2%)** | 0.33 |
| male | VIC | 85 and over | **49238 (1.3%)** | **136 (3.3%)** | 0.14 |

**Note:** As the distribution of participants in the sample is not representative of the distribution of the population of interest (Australians aged 65 years and over), the sample data are weighted to adjust for this, based on gender, state and age. Note that some states have been combined due to small numbers. 15 records were excluded due to missing data on the gender, state or age variables, meaning that the sample *n* = 4172 for weighting calculations.

Weights are calculated for each sample sub-group (sample records from each sub-category of gender, state and age) by the following steps. For each step a worked example is provided with reference to row 1 in Supplementary Table 1. In Step 1 sub-group the proportion (*P*_sub-group_) of the relevant population sub-group represented by the sampled sub-group is calculated by dividing the published population estimate for that subgroup by the number of sample records from that sub-group (Formula 1):

**Formula 1.**


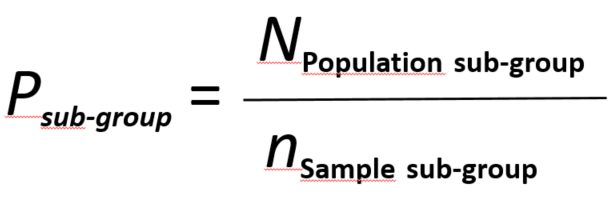


**Worked Example 1.** 85147 / 19 = 4481.42

In Step 2 the sample sub-group weight (*w*) is calculated by multiplying *P*_sub-group_ expressed as a fraction of the summed *P*’s from all sample sub-groups by the total sample *n*_sample_ divided by the sample sub-group *n*_sample sub-group_ (Formula 2):

**Formula 2.**

**
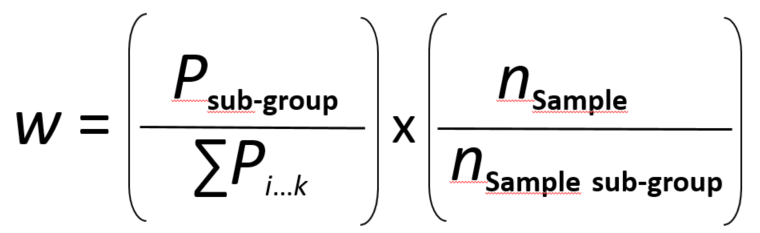
**

**Worked Example 2.** (4481.42 / 80331.43)*(4172 / 19) = 12.25

As these weights indicate, there is generally an underrepresentation of younger males and females, especially in Queensland, and an under-representation of males of any age in the smaller states that have been combined (ACT, TAS, and WA). Victoria and Northern Territory are generally over-represented relative to their populations.

**Abbreviations.** WA: Western Australia, SA: South Australia, VIC: Victoria, NSW: New South Wales, QLD: Queensland, ACT: Australian Capital Territory, TAS: Tasmania, NT: Northern Territory.
